# Harnessing Single Cell Sorting to Identify Cell Division Genes and Regulators in Bacteria

**DOI:** 10.1371/journal.pone.0060964

**Published:** 2013-04-02

**Authors:** Catherine Burke, Michael Liu, Warwick Britton, James A. Triccas, Torsten Thomas, Adrian L. Smith, Steven Allen, Robert Salomon, Elizabeth Harry

**Affiliations:** 1 The ithree Institute, University of Technology, Sydney, New South Wales, Australia; 2 Centenary Institute of Cancer Medicine and Cell Biology, Sydney, New South Wales, Australia; 3 Department of Infectious Diseases and Immunology, The University of Sydney, Sydney, New South Wales, Australia; 4 School of Biotechnology and Biomolecular Sciences and Center for Marine Bio-Innovation, University of New South Wales, Sydney, New South Wales, Australia; University of Massachusetts, United States of America

## Abstract

Cell division is an essential cellular process that requires an array of known and unknown proteins for its spatial and temporal regulation. Here we develop a novel, high-throughput screening method for the identification of bacterial cell division genes and regulators. The method combines the over-expression of a shotgun genomic expression library to perturb the cell division process with high-throughput flow cytometry sorting to screen many thousands of clones. Using this approach, we recovered clones with a filamentous morphology for the model bacterium, *Escherichia coli*. Genetic analysis revealed that our screen identified both known cell division genes, and genes that have not previously been identified to be involved in cell division. This novel screening strategy is applicable to a wide range of organisms, including pathogenic bacteria, where cell division genes and regulators are attractive drug targets for antibiotic development.

## Introduction

Bacterial cell division is essential to bacterial survival, and must be tightly controlled and regulated to ensure the successful generation of two identical daughter cells. This process involves the polymerization of a tubulin-like protein (FtsZ) into a ring at midcell, which then acts as a scaffold for the recruitment of other cell division proteins. These proteins form a complex known as the divisome, which carries out the synthesis and subsequent splitting of the septal cell wall [1]–[3]. The division process must be precisely spatially and temporally regulated to ensure the equal partitioning of DNA into the resulting daughter cells [4]. The essential nature of cell division makes it an attractive target for novel antibiotic development, and several inhibitors of FtsZ are currently under development for this purpose [5]–[8].

Many of the known divisome components have been identified via screens of genetic mutants in the model bacteria *Escherichia coli* and *Bacillus subtilis*. FtsZ, and many other members of the divisome complex (FtsA, FtsN etc.), were identified from temperature sensitive mutations, which result in the formation of filaments (long cells without septa) at the non-permissive temperature [9]. Several of these genes are conserved, to varying degrees, and have subsequently been identified in a wide range of bacteria via gene homology [10]. However, less is known about the regulation of cell division in time and space. Two well known spatial regulators of division site placement, are nucleoid occlusion (Noc) [11], [12] and the Min system [13]. Nucleoid occlusion prevents Z rings forming over the nucleoid or chromosome, while the Min system inhibits the assembly of Z-rings at the cell poles; as the replicated chromosomes segregate, nucleoid occlusion is relieved at midcell allowing formation of a Z ring at this site [14]. But these proteins alone cannot solely account for the regulation of division site placement, as a recent study has shown that in *B. subtilis*, Z rings, while much less frequent, are still positioned precisely at midcell in the absence of Noc and Min [15]. Futhermore, many bacteria do not have clearly identifiable Min or Noc protein homologs, and evidence for other spatial and temporal mechanisms for division site positioning is emerging [16]–[19]. Clearly, the question of how cell division is regulated in bacteria is yet to be completely answered.

Screening for an inhibition of bacterial cell division via gene knockouts or conditional mutations will only identify cell division components that are necessary under standard laboratory conditions, a relatively stress-free and nutrient-rich environment that requires only a small number of essential genes for growth (∼7% for *B. subtilis* and *E. coli*) [20], [21]. This does not reflect the diversity of conditions that bacteria exist and thrive in outside of the laboratory, and many other genes are likely to be required for successful cell division and propagation in their natural environments. For example, *fip*A is required for successful cell division of *Mycobacterium* sp. in the oxidative intracellular macrophage environment [22]. Knowing when, how, and if to divide is essential to a bacterium's ecological success as it faces many environmental stressors. One response to changing environmental conditions is filamentation, which is an inhibition of cell division while the cell continues to grow. This phenotype has been shown to be advantageous in situations including biofilm formation [23], [24], swarming motility [25]–[27], protection from predation [28], [29], resistance to antibiotics [30] and even for successful infection [31], [32]. A wide variety of regulators must therefore exist for responding to environmental cues and controlling cell division, but the molecular mechanisms remain largely unknown. New approaches are necessary for the discovery of these as yet undescribed cell division regulators.

Over-expression of cell division genes and regulators often causes a filamentous phenotype [33]–[35], which is likely to be a result of disrupting the stoichiometry of the interacting divisome components [36]. Overexpression of inhibitors of cell division will also result in a filamentous phenotype as has been shown, for example, for MinC [37], the protease ClpXP [38] and the SOS-inducible SulA [39]. This phenotype has been used to infer a role in cell division for proteins of previously unknown function in *Mycobacterium tuberculosis* [40] and other rod-shaped bacteria [41]. The over-expression of genes in shotgun genomic libraries is therefore likely to result in a filamentous phenotype for clones encoding both known and as yet unknown proteins and regulators of cell division. Additionally, this approach does not rely on particular growth conditions, as over-expressing random genes has the potential to identify cell division regulators that are not normally expressed under standard laboratory conditions.

We present here a method for a high-throughput screen of shotgun genomic expression libraries using flow cytometry analysis and sorting. Our unique flow cytometry screening approach relies only on the different light scattering properties of filamentous versus short (normal-length) cells, and does not require the use of fluorescent dyes, which are often toxic to the cell [42]. We demonstrate the ability to isolate and recover living and reproducibly filamentous clones, allowing for downstream analysis and characterization directly from sorted libraries. We have successfully isolated known cell division genes, and genes which have not previously been described to have a role in cell division for the model organism *E. coli.*, This method can also be applied to a wide range of micro-organisms. The discovery of novel cell division regulators will provide a more complete understanding of bacterial cell biology, and holds potential for the identification of novel drug targets in pathogenic bacteria [7].

## Results

### Discrimination between short and filamentous cells via flow cytometry analysis and sorting

To generate populations of varying cell lengths for the development of a flow cytometry analysis and sorting technique, *E. coli* DH5α cells were treated with the antibiotic cephalexin. Cephalexin inhibits the synthesis of peptidoglycan at the division septum in *E. coli*, resulting in filamentous cells [43]. The average cell length of DH5α cells during exponential growth without cephalexin was 2.89 µm (±1.38), ranging from a minimum of 1.34 µm to a maximum of 15.05 µm as determined by phase contrast microscopy. The vast majority of cells (94.98%±2.89) were less than 5 µm in length, and 4.05% (±2.99) were between 5 and 10 µm. A small proportion of the population, 0.96% (±0.95) was observed to be filamentous, defined here as >10 µm in cell length. The addition of cephalexin to DH5α cultures resulted in longer average cell lengths, which increased with exposure time to cephalexin. Average cell length increased to 6.05 µm (±2.95) at 1 hour, 11.13 µm (±4.63) at 1.5 hours, 27.62 µm (±14.87) at 2 hours and 42.46 µm (±15.72) at 2.5 hours exposure time (Figure S1).

**Figure 1:**
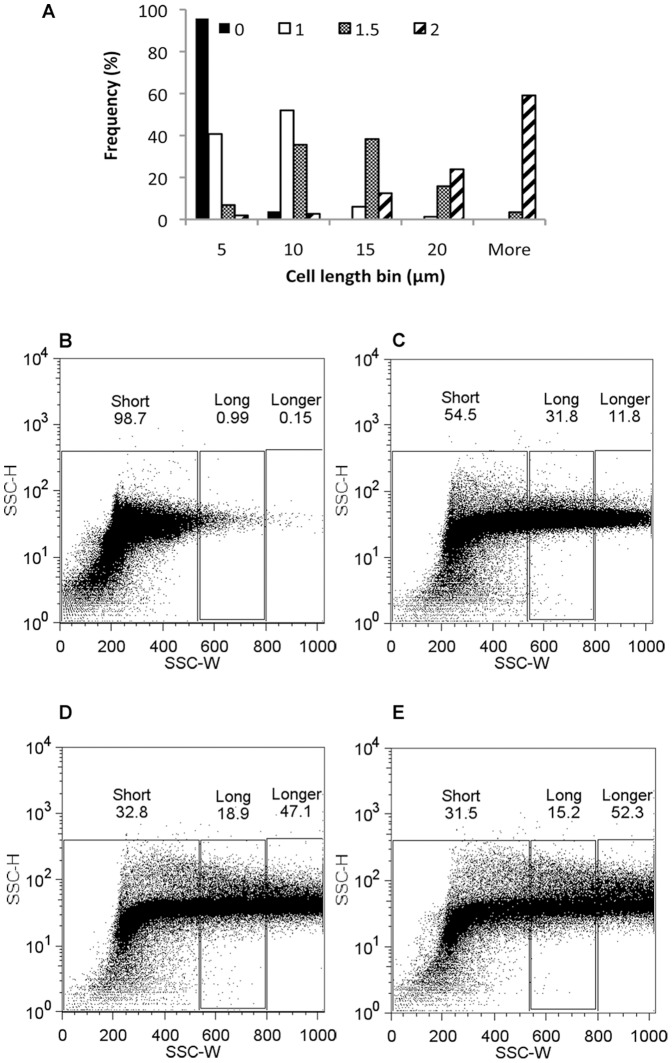
Flow cytometry analysis of *E. coli* populations of varying cell lengths. (A) Cell length distributions for *E. coli* DH5α populations either not exposed to cephalexin (0) or exposed to cephalexin for 1 hour (1), 1.5 hours (1.5) or 2 hours (2). (B–E) Flow cytometry analysis of the corresponding populations displayed as dot plots with SSC-H plotted against SSC-W. (B) Not exposed to cephalexin, (C) 1 hour exposure, (C) 1.5 hours exposure, (D) 2 hours exposure. The percentage of events in each gate for each population is displayed at the top of each gate, 100 000 events from each population are displayed.

These populations of increasing average cell length were fixed, and subsequently used for flow cytometry analysis. A trend of increasing side scatter width (SSC-W) signal with increasing cell length was observed, as seen in Figure 1. The distribution of cell lengths for each population is shown (Figure 1a) along with the corresponding dot plots (Figure 1, b–e), where each dot represents a single cell or event from the population. Populations with a greater proportion of filamentous cells (>10 µm cell length) contained an increasing proportion of events with increased SSC-W values.

**Figure 2:**
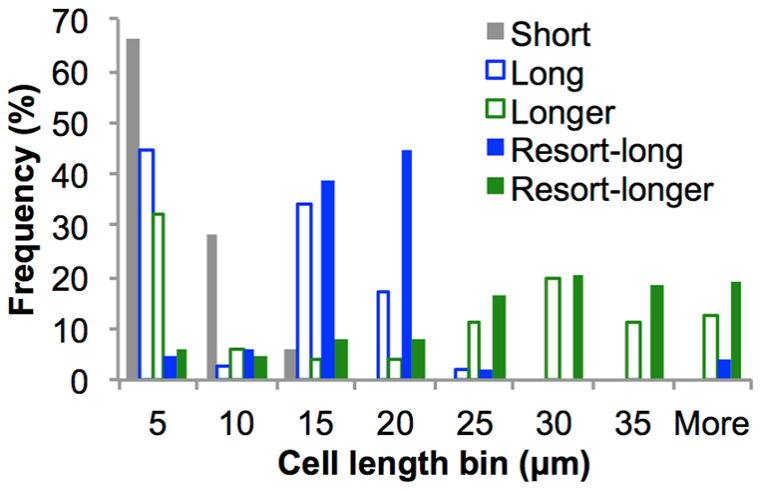
Cell length distributions of sorted populations. Sorted from the gates “short”, “long” and ”longer as defined in Figure 1. Long and longer sorted populations were re-sorted from their respective gates to yield the resort-long and resort-longer populations. Cell lengths were measured via phase contrast microscopy.

We confirmed that increasing cell length does correlate to increasing SSC-W by sorting cells from a mixed population encompassing a range of cell lengths. The populations of fixed cells described above were combined, and sorted on the basis of increasing SSC-W (gates as shown in Figure 1). Additionally, sorted populations from the “long” and ”longer” gates were resorted from the same gate, applying more stringent conditions for purity of the sorted populations. Sorted populations were examined using phase-contrast microscopy, which revealed that the population sorted from the gate with the smallest SSC-W values (short) was made up predominantly of non-filamentous cells of less than 10 µm in length, while populations sorted from gates with increasing SSC-W values (“long” and “longer”) were enriched for filamentous cells (>10 µm) (Figure 2). Re-sorting removed a large proportion of contaminating short cells from the “long” and “longer” sorted populations, decreasing their proportion from 47.2% (long) and 37.7% (longer), to 10.5% (long) and 10.6% (longer) in the resorted populations.

**Figure 3:**
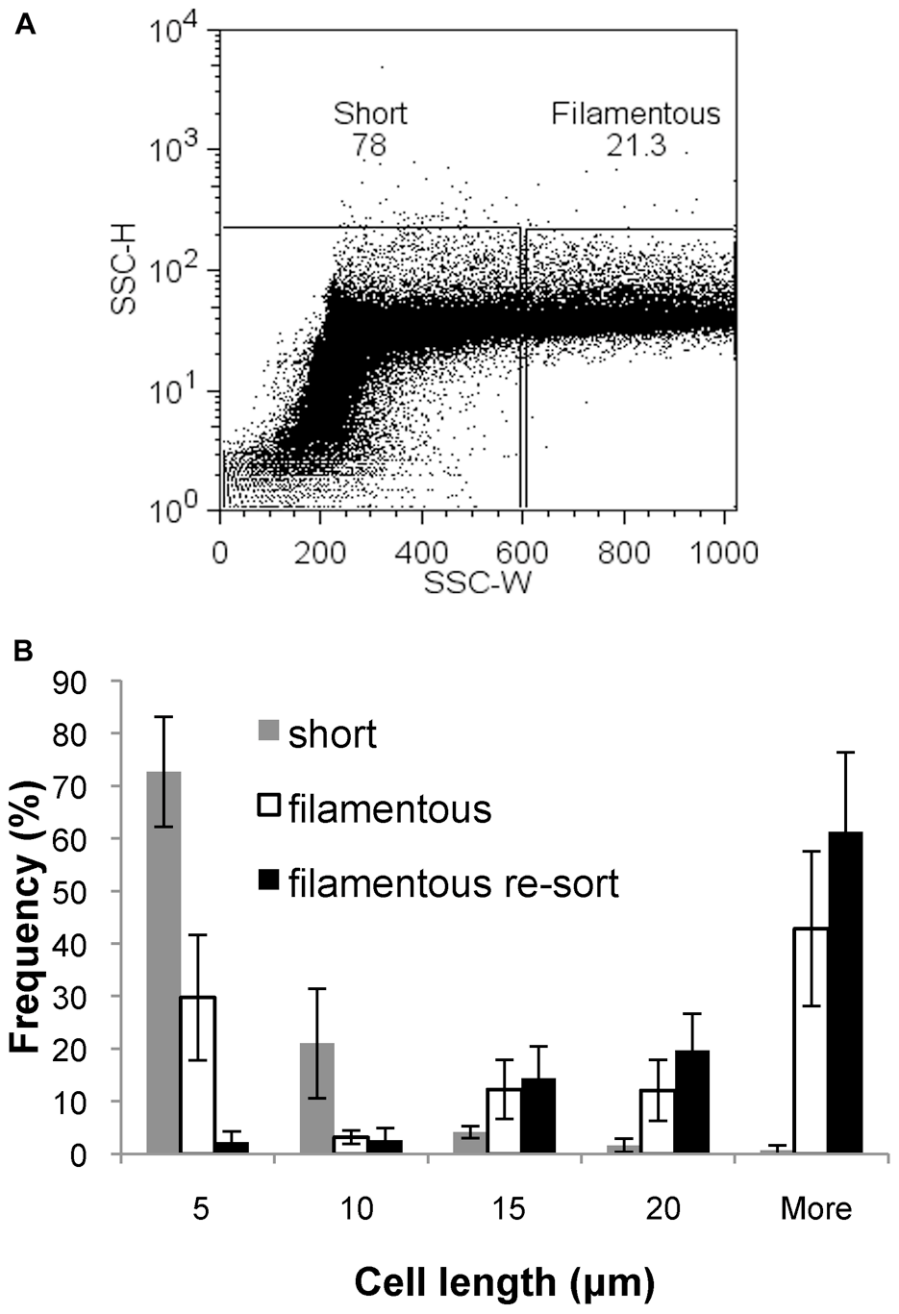
Filamentous cells are effectively sorted from mixed populations by flow cytometry sorting. (A) Mixed populations of various cell lengths were sorted from “short” and “filamentous” gates, and re-sorted from the filamentous gate. (B) Cell length distributions of populations sorted from “short” (grey bars), and “filamentous” (open bars) gates, and re-sorted from “filamentous” gate (black bars). Data was collated from 2 independent sorts each of fixed and live cells.

For subsequent sorting experiments, we took the approach of defining two gates, “short” and “filamentous”. The “short” gate was created to encompass greater than 99% of a non-cephalexin treated DH5α population, and the “filamentous” gate encompassed the same area of the SSC-H axis, and all SSC-W values greater than the “short” gate (Figure 3). Sorting was carried out on mixed populations (cephalexin treated as described above) of both fixed and live cells. Live cells were formaldehyde fixed immediately post sorting or re-sorting to preserve the phenotype for downstream microscopy and analysis. Microscopic analysis of sorted and re-sorted populations gave similar results for both live and fixed cells, and the separation of short and filamentous cells was consistent and reproducible. Populations sorted from the “short” gate contained more than 90% short cells (<10 µm length), and re-sorted populations from the “filamentous” gate contained more than 90% filamentous cells (>10 µm length), as shown in Figure 3. Therefore, filamentous cells are able to be effectively isolated from mixed populations using a flow cytometry sorting approach.

### Isolation of live, reproducibly filamentous clones from a mixed population via flow cytometry sorting

The previous results showed that filamentous cells could be efficiently recovered from mixed populations, however it was not known whether these cells were still viable after sorting. Cephalexin treated cells could not be used for this purpose, due to cephalexin's effect on cell viability. Additionally, those cells which do survive cephalexin exposure and sorting would revert to a short cell phenotype after the removal of cephalexin, making it impossible to distinguish what proportion of the resulting viable cells were short versus filamentous at the time of sorting. To test this, strain EC766 (ftsZ*, P_BAD_) was created for the controlled induction of filamentous cells via overexpression of the *fts*Z gene. EC766 (ftsZ*, P_BAD_) contains a second copy of *fts*Z cloned into the pBAD24 vector, under control of the arabinose inducible P_BAD_ promoter [44]. Overexpression of *fts*Z by 10 fold or more causes a block in cell division, and results in filamentous phenotype [45]. Three hours of arabinose induction of EC766 (ftsZ*, P_BAD_) resulted in filamentous cells, as shown in Figure S2.

**Figure 4:**
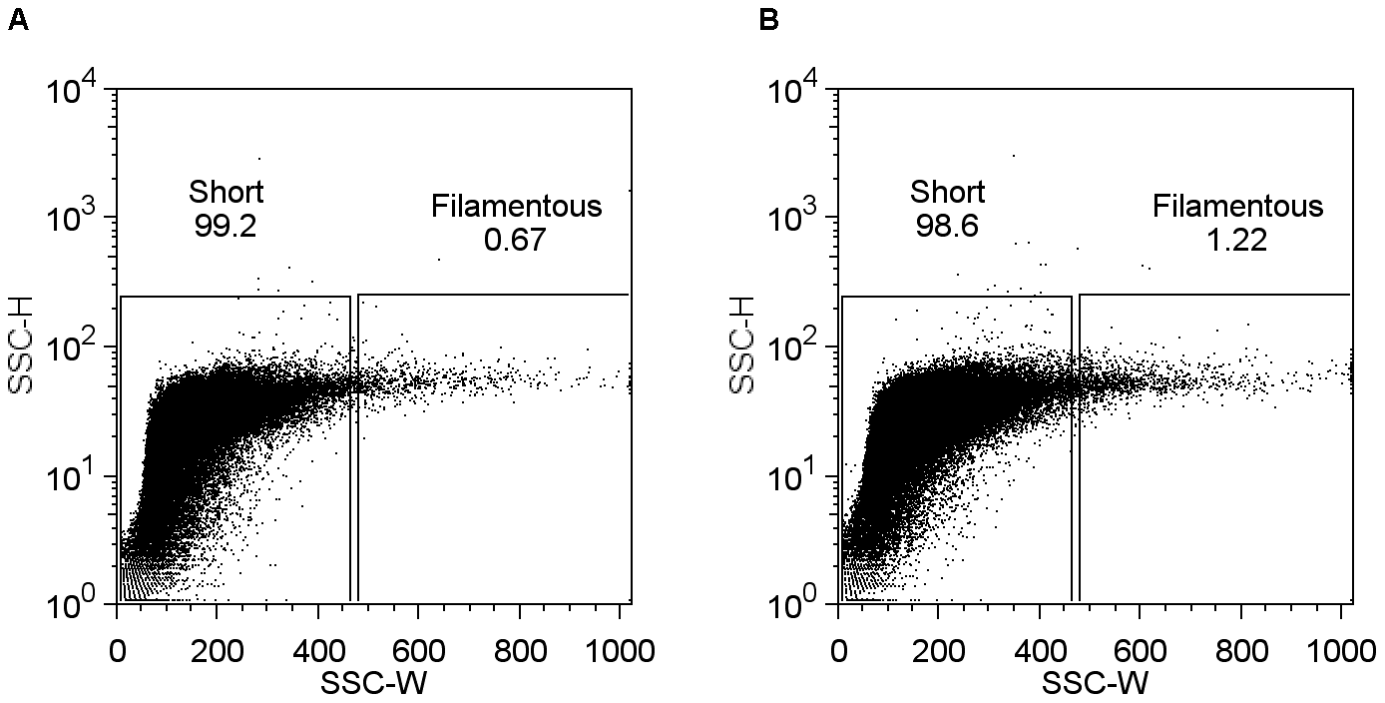
Detection of filamentous clones in a mixed population. Dot plots of (A) control population (EC764, short cells) and (B) a mixed population of predominantly short cells (EC764) spiked with filamentous cells (induced EC766 (*fts*Z*, P_BAD_). Numbers at the top of each gate represent the percentage of events contained within that gate. An increase in events (filamentous cells) is observed in the “filamentous” gate between the control and mixed populations.

A population of mixed cell lengths was created by spiking a short cell control culture, *E. coli* EC764 (DH5α pBAD24, no insert), with filamentous (arabinose induced) *E. coli* EC766 (ftsZ*, P_BAD_). Filamentous cells were isolated from the mixed by population by sorting and re-sorting from the “filamentous” gate (Figure 4). Colony PCR of 30 randomly selected colonies yielded 15 PCR products corresponding to the cloned *fts*Z gene, indicating that half of the colonies obtained were filamentous EC766 (ftsZ*, P_BAD_) clones from the mixed population. This result is consistent with the dot plots of the control (EC764) and mixed populations (Figure 4) which show an increase from 0.65% (control) to 1.10% (mixed) of events in the “filamentous” gate, indicating that approximately 40% of “filamentous” events in the mixed population were due to the spiked EC766 (ftsZ*, P_BAD_) filamentous cells. Colonies obtained from the screen that did not contain the cloned *fts*Z gene were therefore assumed to be a result of the small fraction of naturally occurring filamentous cells from the control population.

Despite the recovery of non-target events, the screen was successful in isolating living, reproducibly filamentous clones, even though they represented only a small fraction of the mixed population.

### Assessment of viability during flow cytometry sorting and re-sorting

In the experiment described above, culturing of 3000 resorted events resulted in 142 colonies, indicating that only 4.7% of sorted events survived the screen. To optimize cell viability during flow sorting, the handling of cells prior to, during and post sorting was altered. Changes to the screening protocol, including sorting from and into M9 media rather than PBS and avoiding storing cells on ice, were successful in increasing the viability of cells post sorting. Of 3000 re-sorted events collected, 2901 (short re-sort) and 1361 (filamentous re-sort) colonies grew after culturing, indicating that 96.7% of short re-sorted cells and 45.4% of filamentous resorted cells were viable, as compared to 4.5% viability under the previously used conditions.

We also measured the viability of sorted and re-sorted cells using live/dead staining, to determine which stage of the sorting process affected cell viability. Live/dead staining indicated that the vast majority of the mixed population was viable before sorting, with 95.7% of events staining as live, and 0.5% of events staining as dead. The remaining events were unstained, possibly representing inanimate particles or electronic noise. Considering only the events that stained with either live (SYTO9) or dead (propidium iodide) stain, analysis of sorted populations indicated that the majority of cells remained viable after one sort with 98.8% (short sort) and 89.6% (filamentous sort) live cells. The proportion of live stained cells decreased after a second sort to 41.6% (short re-sort) and 42.7% (filamentous re-sort). The proportion of the re-sorted filamentous population which stained as “live” is similar to the proportion of sorted events which yielded colonies with culturing (45.4%, see above). However, this is not the case for the short re-sorted cells, which displayed decreased viability with the staining method (41.6%) compared to culturing (96.7%, see above).

These results indicate that successive sorting does decrease cell viability for filamentous cells. Nevertheless, the high throughput nature of this screen means that many viable, filamentous clones can still be obtained by optimizing the sorting conditions. As such, the isolation of large numbers of live, reproducibly filamentous clones from mixed populations is feasible with this approach.

### Creation of a shotgun expression library from *Escherichia coli*

Shotgun expression libraries were created from the model bacterium *E. coli* with the aim of screening for inhibitors and regulators of cell division. The cloning strain DH5α was used, which contains a mutation in the RecA gene (recA1). This mutation renders the RecA protein inactive, thereby preventing induction of the SOS response [46], [47]. Approximately 61 500 clones (pBAD24, arabinose inducible expression vector) were obtained for the *E. coli* DH5α library. Colony PCR indicated that the majority of inserts were between 1–3 kb (∼93%), with a small proportion of inserts >3–5 kb (∼7%). High genomic coverage was achieved with at least 20× coverage and >99% probability of capturing any given genome fragment.

### Screening for filamentous clones from a shotgun expression library and confirmation of filamentous phenotype

The *E. coli* library was induced for over-expression of cloned DNA fragments by arabinose. The induced library was compared via flow cytometry to a control culture EC764 (pBAD24), to determine whether an increase of events in the “filamentous” gate (i.e. SSC-W values larger than >99% of the control population) was observed. For induction at 0.02% arabinose, only a small increase of events in the “filamentous” gate was observed from 0.39% of the total population in the control, to 0.40% in the *E. coli* library (increase of 2.5%). For induction at 0.2% arabinose, a larger increase was observed, from 0.26% of the total population in the control, to 0.38% in the *E. coli* library (increase of 46.2%). The culture induced at 0.2% arabinose was therefore screened for filamentous cells, using the approach described above.

**Figure 5:**
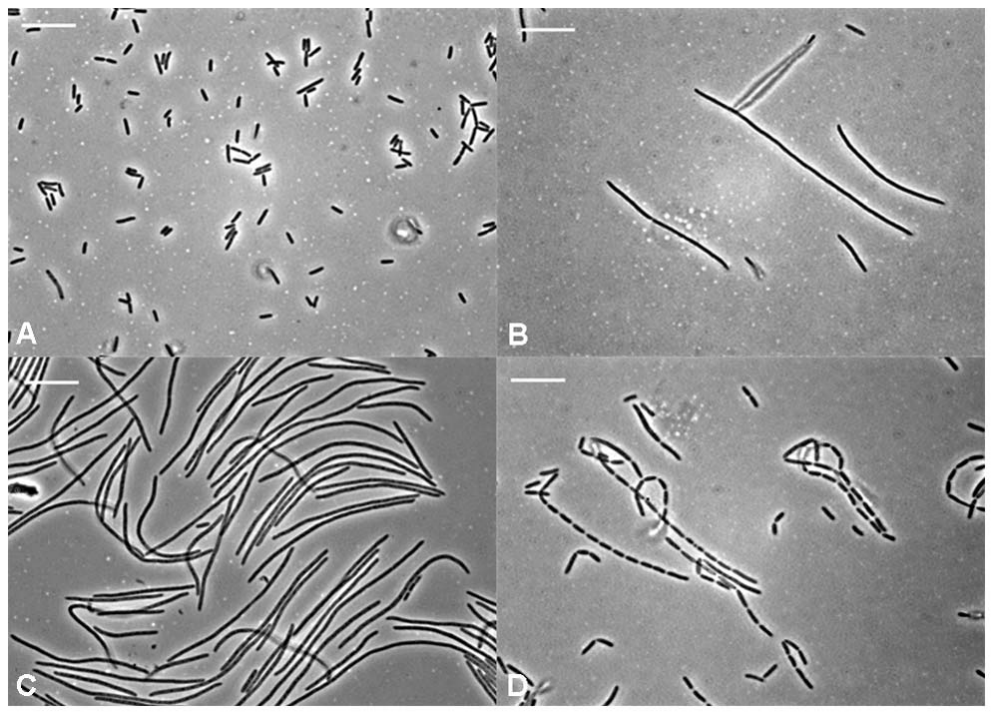
Representative images of control cells and clones with a filamentous phenotype isolated from the screen. (A) Control cells of strains EC766 (DH5α, pBAD24). (B–D) examples of phenotypes of *E. coli* clones isolated from the screen. Different degrees of filamentation were observed, along with a chaining phenotype as shown in (D). Scale bar = 10 µm.

More than 500 colonies were obtained from the screen, and 160 were randomly selected for re-induction and confirmation of filamentation via microscopy. 45 clones (28.1%) displayed a reproducibly filamentous phenotype under induction, examples of which are shown in Figure 5.

Clones with a chaining phenotype were not considered further, as we were primarily interested in inhibition of cytokinesis, rather than a defect in cell separation. The remaining clones (n = 23) were re-tested for filamentation with induction to confirm the phenotype. After removing those clones that did not filament on re-induction, 22 clones were considered to be reproducibly filamentous.

### Assessment of false positive rate for induced libraries

**Figure 6:**
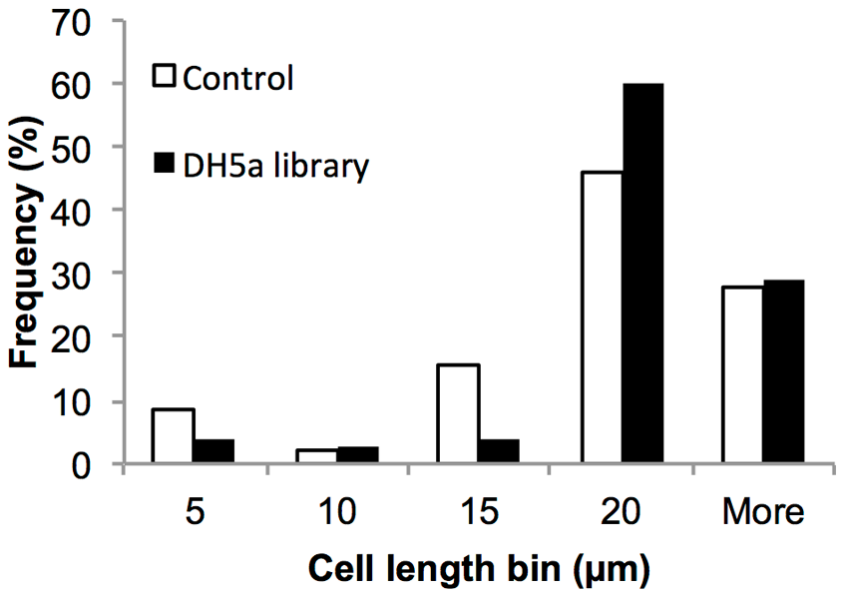
Cell length distribution of re-sorted filamentous cells from induced control EC764 (DH5α, pBAD24) and DH5α genomic library populations. Data based on cell length measurements from 96 (control) and 75 (DH5α library) cells.

Despite the fact that very few contaminating short cells were detected in re-sorted populations from test sorts of fixed cells (4.7%±4.4), much higher rates of false positives were observed when screening the genomic libraries. Seventy-two percent of colonies obtained from the *E. coli* screen were false positives (i.e. were not filamentous on re-induction from the P_BAD_ promoter). To confirm that the screen does isolate highly enriched populations of filamentous cells, induced populations of both the control EC764 (pBAD24), and the DH5α genomic library were screened for filamentous cells as described above, except that re-sorted cells were fixed immediately post-sorting. Microscopic analysis of re-sorted populations from the “filamentous” gate confirmed that the majority of cells captured in the screen were indeed filamentous at the time of sorting (Figure 6). Filamentous cells were also recovered from the control population (EC764), representing the small proportion of cells in the host, *E. coli* DH5α, which are naturally filamentous during exponential growth (for unknown reasons), but are not reproducibly filamentous with over-expression of the cloned insert DNA. This suggests that at least some of the false positives obtained from the library screens result from the small proportion of naturally occurring filamentous host cells. This assumption is supported by the flow cytometry data, where the percentage of events in the filamentous gate for the control (0.26% of the total population) accounts for 68% of the total events in this gate for the induced library (0.38% of the total population), which is similar to the rate of false positives observed (72%).

### Identification of genes involved in cell division

**Figure 7:**
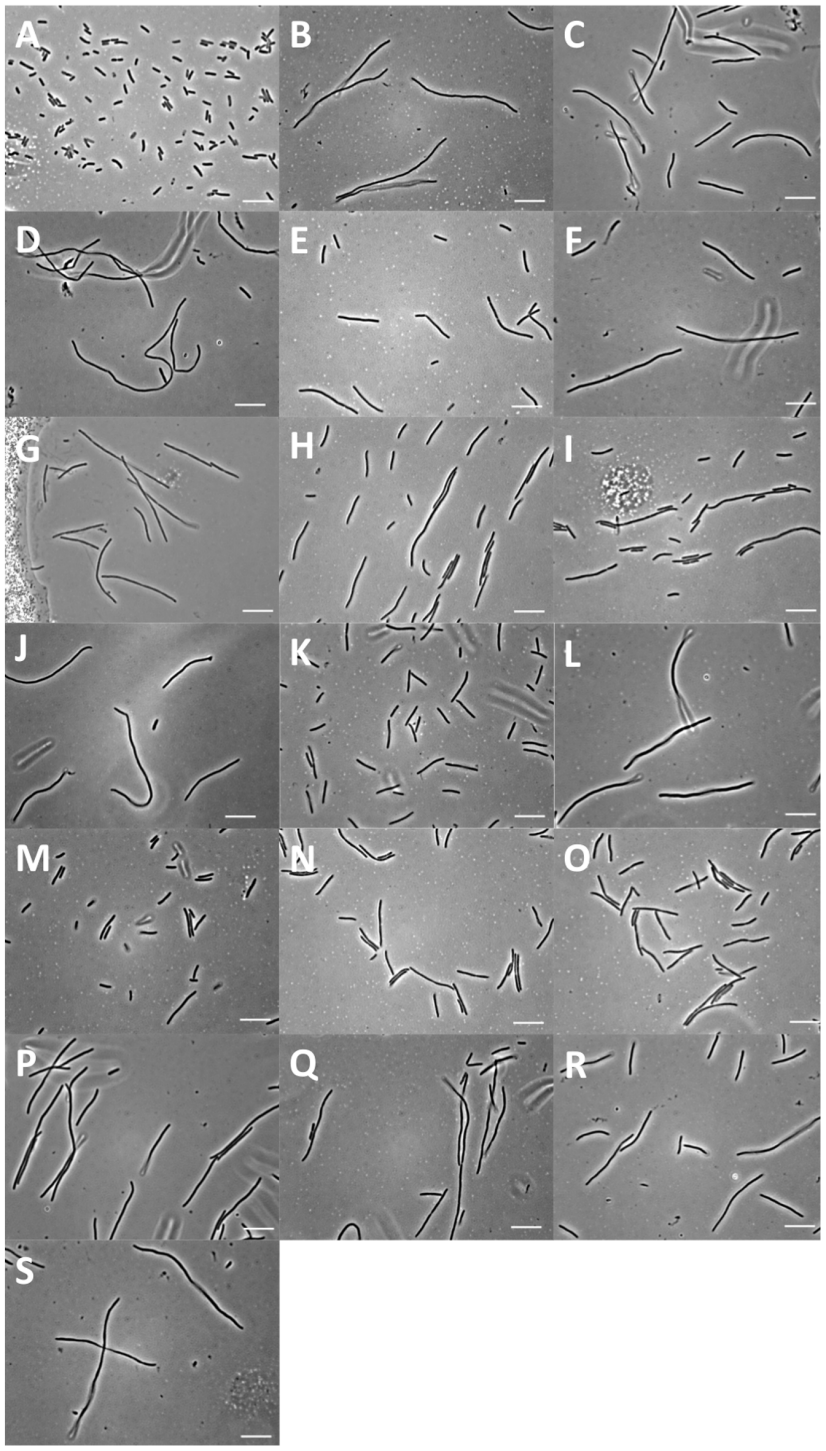
Representative images of the phenotype observed for each clone listed in Table 1 with arabinose induction. (A) Negative control EC764, (B–D) clones containing the aroB-dam geneic locus, (B) D2–3D, (C) D2–8G, (D) D2–4B. (E) D1–2H encoding *yej*H. (F) D2–3B and (G) D2–7H encoding *ycj*Y. (H) D2–5G and (I) D2–7D encoding *ytf*A-B. (J) D2–10F encoding *rpl*L-*rpo*B. (K) D1–8E encoding a region of the e14 prophage element. (L) D2–8D and (M) D1–9C encoding a region of the CP4-6 prophage element. (N) D2–7F and (O) D2–8F encoding a portion of the histidine biosynthesis operon. (P) D1–5C encoding the Kil protein from the Rac prophage element. (Q) D1–5F and (R) D2–11E encoding two distinct peptidylprolyl-cis-transisomerase genes *ppi*A and *ppi*C respectively. (S) D1–9G encoding *mut*T. Images taken using phase contrast, scale bars = 10 µm.

Clone inserts from the 22 clones with a reproducibly filamentous phenotype were sequenced to determine which regions of the *E. coli* genome were represented. Sequences aligned to 12 distinct sites in the *E. coli* genome, including 11 in the *E. coli DH1* genome (the DH5α genome is not publicly available), along with an additional site from the *E. coli* DH10B genome, which is not present in the DH1 genome (Table 1). These regions contain both known cell division genes and genes that have not previously been identified to be involved cell division. Table 1 lists the 12 genetic loci, and the genes contained in each of the isolated clones, along with the average cell length of each clone under inducing conditions, and the proportion of the population which was filamentous (i.e. >10 µm cell length). The degree and rate of filamentation for each clone varied, and the cell length distribution for each was significantly different (P<0.05) to the control (EC764), as assessed by a two tailed t-test. Figure 7 shows representative images for each clone as examples of the filamentous phenotypes obtained. One clone from each loci was transferred to K12 MG6155 [48], a standard reference strain, and tested to ensure that filamentation was a result of the cloned DNA, and not a mutation on the host cell chromosome. Similar levels of filamentation were observed for all clones.

**Table 1:** Genetic loci, genes and phenotypes for filamentous clones.

Genetic loci	Clone	Genes present	Cell length µm (S.D.)	Frequency of cell lengths >10 µm (%)
aroB-dam	D2–3D	*aro*B (273–362/362)	22.8 (15.8)	75.6
		*dam*X		
		*dam* (1–240/278)		
	D2–8G	*aro*B (276–362/362)	15.7 (7.9)	76.6
		*dam*X		
	D2–4B	*aro*B (273–362/362)	24.8 (16.3)	78.1
		*dam*X		
yejH	D1–2H	*yej*H (1–461/573)	10.0 (8.2)	37.5
ycjY	D2–3B	*ycj*Z (207–1/299)*	23.2 (20.6)	69.9
		*ycj*Y		
	D2–7H¥	*ycj*Y (6–306/306)	18.9 (9.6)	80.2
				
ytfA-ytfB	D2–5G	*ytf*B (34–212/212)	10.7 (4.6)	51.7
		*ytf*A (108–31/108)*		
	D2–7D	*ytf*B (37–212/212)	8.6 (5.0)	30.4
		*ytf*A (108–23/108)*		
rplL-rpoB	D2–10F	*rpl*L	34.5 (21.1)	93.1
		*rpo*B (1–1227/1342)		
Prophage e14	D1–8E	*ymf*L (142–185/185)	8.9 (4.1)	33.0
		*ymf*M		
		*ymf*N (1–92/455)		
Prophage CP4-6	D2–8D	*yaf*W (85–105/105)	23.7 (10.3)	93.4
		*ykf*I		
		*thr*W*		
	D1–9C	*ykf*H (10–73/73)	5.2 (2.2)	3.7
		*yaf*W		
		*ykf*I		
hisB-hisA	D2–7F	*his*B (226–355/355)	11.3 (4.1)	54.4
		*his*H		
		*his*A (1–234/245)		
	D2–8F	*his*B (226–355/355)	16 (11.8)	62.86
		*his*H		
Rac prophage≠	D1–5C	*kil*	19.4 (9.1)	89.9
ppiA	D1–5F	*ppi*A	13.9 (11.2)	44.76
ppiC	D2–11E	*ppi*C	16.4 (8.8)	69.9
		*ilv*C (198–491/491)*		
mutT-coaE	D1–9G	*mut*T (43–129/129)	47.7 (22.3)	100
		*yac*G*		
		*yac*F*		
		*coa*E*		

Genomic regions identified by DNA sequencing of reproducibly filamentous clones. The individual genes present in each clone are listed. Where open reading frames were partially cloned, the amino acid positions present are indicated in brackets, followed by the total number of amino acid residues for the full ORF after the backslash. The average cell lengths of induced clone populations are indicated, based on measurements of at least 100 cells, along with the percentage of each population that was filamentous (i.e. >10 µm cell length).

* denotes genes which are cloned in the opposite orientation relative to the expression promoter.

¥ This clone was identified five times.

≠ This genomic region is not present in the DH1 genome, but was identified from the DH10B genome.

## Discussion

We have developed a method for the identification of novel bacterial cell division proteins and regulators using shotgun over–expression of genomic libraries and flow cytometry sorting. Using only the light scattering properties of bacterial cells, we applied flow cytometry sorting to screen a large genomic library, screening and sorting millions of cells in less than one hour. This single-pass screen resulted in the isolation of several known and also putative novel regulators of cell division, and the short time frame from screen to verification allows for the screening of genomic libraries under several different inducing conditions, which would likely identify additional division genes.

In order to successfully isolate filamentous cells from mixed populations using flow cytometry, we first had to determine which parameter would allow us to distinguish between short and filamentous cells. The observation that cell length corresponds to SSC-W signal is consistent with the fact that pulse width is proportional to the period of time that it takes for a particle to move through the laser interrogation point in a flow cytometer [49]. Assuming that hydrodynamic focusing of the sample results in a rod-shaped cell moving through the interrogation point in a straight line from one cell pole to the other, the width of the signal pulse obtained should be proportional to the length of the interrogated cell. This means that the height of the laser spot used to interrogate the cells will be related to resolution of different cell lengths. Indeed we did observe differences in population distributions (SSC-H vs SSC-W) when comparing identical populations on flow cytometers with different focus spot heights (data not shown), although many other differences between specific machines and experimental set-ups (e.g. stream velocity) could also contribute to differences. This highlights the importance of the specific optics, electronics and configuration of any particular sorting flow cytometer, and is an important consideration for reproducing the results described here. In the case of the Aria II flow cytometer used here, cells of >10 µm in length could consistently be isolated from mixed populations.

Flow cytometry sorting of bacteria based on cell size has only once been previously reported in the literature. Fluorescence pulse width was used to sort extremely filamentous cyanobacteria, utilizing naturally occurring fluorescent pigments in the cyanobacterial cell [50]. However, most bacteria do not naturally fluoresce, and fluorescent stains frequently have an adverse effect on cell viability [42], making them unsuitable for the recovery of live cells for further characterisation. Our aim was to avoid the use of fluorescent stains, relying only on the light scattering properties of bacterial cells, and we successfully applied cell sorting based on the parameter of SSC-W to isolate filamentous cells from mixed populations. We found FSC-W to be another useful parameter to sort filamentous cells, but SSC-W gave a more sensitive discrimination of cell length (data not shown).

Re-sorting of the target population was essential to reduce the proportion of non-target cells obtained in sorted populations, which may be a result of the sorting masks used. Sorting masks define how droplets are deflected for sorting, and can be altered to increase yield or purity. A yield mask increases sorting yield, but can result in additional droplets being sorted along with the droplet containing the target event. A purity mask ensures a highly pure population by only sorting droplets containing target events, but at the expense of recovery and yield [51]. We attempted to obtain a more pure population in the first sort by employing a purity mask from the outset, but found this to be impractical for sorting large numbers of cells, as the efficiency of sorting was decreased, and the time it took to sort the same number of target events was increased (data not shown). Furthermore even when using a purity mask the final purity is limited by fact that sorting on a single positive parameter (in this case SSC-W) means that coincident events consisting of a long and short bacteria will always be classified as “long” (in effect the short particle will be invisible to the sorter if it is coincident with the long particle), resulting in contamination of the sorted fraction with some amount of short particles. This effect can be reduced by diluting the sample and slowing down the sort rate, but again this dramatically increases the total time required for the sort. The approach of using a yield mask for the initial sort, followed by re-sorting of smaller, more dilute samples, with a more stringent purity mask increased the overall speed and efficiency of the sorting process.

Apart from the toxic effects of fluorescent stains, the physical conditions encountered by cells in the flow cytometer may also affect cell viability, such as pressure applied in the sheath fluid, exposure to the laser, effects of the voltage applied at the deflecting plates for sorting, and impact on the surface of the collection vessel [42]. We achieved viabilities of re-sorted filamentous cells of up to 45%. This was in agreement with live/dead staining which indicated a decrease in cell viability to 42.7% following a second round of cell sorting. While culturing of short re-sorted cells did not correspond to the staining results, these results did indicate that short cells have a higher degree of viability following the screening process. It is possible that short cells are physically more robust than filamentous cells to the pressures encountered during the sorting process. In any case, given the high-throughput nature of the screen, recoveries of 45% of viable filamentous cells are more than sufficient to carry out a comprehensive screen.

It is also possible that forcing the overexpression of cloned genes that cause filamentation affects the viability of these clones. The P_BAD_ promoter is not dose responsive at the single cell level [52], however more favorable expression conditions could be optimized with the use of a range of inducer concentrations and alternative host vector systems [53].

Our single-pass, proof-of-concept screen was highly successful, yielding 22 clones with inducible, reproducible filamentous phenotypes, and which aligned to 12 distinct loci within the *E. coli* genome. Genes with both known and unknown roles in cell division were identified. Genes previously identified to be involved in cell division include *dam*X which is known to inhibit cell division when overexpressed [54]. DamX has recently been shown to interact with the essential division protein FtsQ and is thought to contribute to cytokinesis [55]. Null mutations of *dam*X render *E. coli* sensitive to bile salts, suggesting that *dam*X is important for survival in the gut environment [56]. Other genes identified in this screen which have previously been identified to have a role in cell division include genes from the histidine biosynthesis operon [57], and the Rac prophage-enocoded Kil protein [58].

We also identified genes within the lambdoid prophage element e14, *yfm*M and *yfm*N. *yfm*M has no known function, while *yfm*N is thought to be a fusion of a replication protein and a phage terminase protein [59]. A region of the e14 element including ymfM-N has been associated with cell death [59], [60], and an SOS-inducible inhibition of cell division has also been associated with the e14 prophage [61]. These previous studies suggested that inhibition of cell division occurred via inhibition for FtsZ polymerization. As such it is possible that one of the genes captured here acts as a direct inhibitor of FtsZ.

We identified genes that have not previously been reported to be involved in cell division. Two distinct clones from the *E. coli* library mapped to the gene *ycj*Y. This gene encodes an uncharacterized protein that is annotated as a predicted hydrolase, based on its similarity to COG1073, a family of hydrolases. Enzymes which hydrolyse peptidoglycan, a component of the bacterial cell wall, are essential for remodeling of the cell envelope for cell growth and division [62], and have also been shown to modulate cell shape for increased attachment to the host in pathogenic bacteria [63]. It is possible that *ycj*Y plays an as yet unidentified role in *E. coli* cell division, and investigations are currently underway to elucidate how over-expression of this gene acts to inhibit cell division in *E. coli*.

The CP4-6 prophage-encoded toxin/antitoxin system *ykf*I/*yaf*W was also identified in this screen. The YkfI toxin has been reported to be toxic during overexpression in *E. coli*, however its effect on cell division has not previously been described. It is interesting to note that the clone encoding the full toxin/antitoxin pair (clone D1_9C) has a much less severe phenotype than the clone with a full toxin gene (*ykf*I), but only a partial antitoxin gene (*yaf*W) (clone D2_8D) (Table 1). Other genes which have not previously been reported to have a role in cell division include *ytf*B encoding an uncharacterized protein, the nucleoside triphosphate phosphohydrolase gene *mut*T, and genes encoding two distinct peptidylprolyl-cis-transisomerases, ppiA and ppiC. The role of these genes or gene products in inhibiting cell division when over-expressed is currently unknown, and is the subject of ongoing investigations.

The screen was successful in identifying novel candidate genes involved in bacterial cell division and regulation, but a high rate of false positives was encountered. We wanted to identify the likely cause, so that future screens could be optimized. Microscopic analysis of screened *E. coli* library cells fixed immediately after re-sorting confirmed that more than 90% of captured cells are filamentous at the time of sorting, suggesting that a large portion of the false positives obtained were filamentous for reasons other than over-expression of the cloned genes. Screening of a control population containing only cells with an empty vector, confirmed that the small proportion of naturally filamentous cells observed in the host strain are efficiently captured with this highly sensitive method. A small proportion of filamentous cells were always observed in the host DH5α populations examined. Variable rates of filamentation were also observed in other *E. coli* strains, ranging from low levels (comparable to DH5α) in *E. coli* JM109, to much higher levels in *E. coli* BL21(DE3) (data not shown), making the latter strain an unsuitable host for this screening application. The cause of this spontaneous filamentation in these *E. coli* strains is unknown, but may possibly be due to induction of the SOS response due to cell stress or DNA damage [39].

As such, some degree of false positives can always be expected where even a small proportion of the host strain population naturally filaments. A reduction in the rate of false positives could be achieved with the use of alternative host strains with a lower level of natural filamentation, or alternatively by improving the ratio of natural host filamentation to induced filamentation through optimization of induction conditions. However, even where the rate of occurrence of target events is low, as is often observed in large scale screening studies (less than 0.4% of the population in this case), the power of this approach lies in the ability to efficiently isolate those rare events from large populations.

## Conclusions

We have developed and validated an approach for the discovery of novel cell division proteins and regulators. The use of random genomic over-expression libraries is an alternative to more traditional approaches of genetic mutation and gene knockouts, and the use of flow cytometry allows for a high throughput screening of many thousands of clones under many different conditions. The utility of this approach has been demonstrated here through the identification of both known and novel putative cell division proteins in the model bacterium *E. coli*, and this is amenable to a wide variety of bacterial species. Improvements in the optics and resolution of flow cytometry sorters currently available should allow for even greater differentiation of cells based on size; for example, the BD Influx has reported resolutions of 0.2 µm, and the ability to adjust the spot height of the laser beam. This will open the door to many types of screens based on cell shape and size. An understanding of the regulators of these processes will provide novel insight into the ecology of bacteria in different environments, and holds great potential for the identification of new targets for the development of novel antimicrobials.

## Methods

### Bacterial strains and growth conditions

Bacterial strains used in this study are listed in Table 2. *Escherichia coli* DH5α and K12 MG1655 were maintained and grown on LB media (BD Biosciences, San Jose, CA, USA). For strains containing the pBAD24 plasmid 100 µg/ml ampicillin (Sigma-Aldrich, St Louis, MO, USA) was added to the media. Liquid cultures were always incubated at 37°C and 150 rpm, and agar plates were incubated statically at 37°C.

**Table 2:** Bacterial strains used in this study.

Strain	Description	Source
*E. coli* DH5α	Cloning strain	New England Biolabs
*E. coli* K12 MG1655	Wild-type strain	ATCC
*E. coli* EC764	*E. coli* DH5α, pBAD24 vector	This study
*E. coli* EC766	*E. coli* DH5α, *fts*Z gene cloned into the pBAD24 vector	This study

Over-expression experiments with the pBAD24 vector were carried out in M9 media [64] containing 0.4% glycerol and 0.1% casamino acids (w/v). Overnight cultures were used to inoculate M9 media to an initial OD_600_ of ∼0.05. Cultures were incubated for two hours, followed by the addition of arabinose (inducer) to 0.02 or 0.2% (w/v), followed by incubation for a further 3–4 hours. Where indicated, glycerol was replaced with 0.4% glucose for repression of expression from P_BAD_.

Strain EC766 (ftsZ*, P_BAD_) was created for the controlled induction of filamentous cells via overexpression of the *fts*Z gene under control of the arabinose inducible P_BAD_ promoter. EC766 was constructed by amplifying *fts*Z from plasmid pKD3 [45] (kindly provided by Jo Luktenhaus) by PCR using primers ECftsZ_F and ECftsZ_R (Table 3) with *Pst*I and *Xma*I sites incorporated PCR was carried out with Phusion Taq (NEB, Ipswitch, MA, USA) under standard conditions. Vector pBAD24 and the *fts*Z PCR product were digested with *Xma*I and *Pst*I (NEB) and ligated with a T4 DNA ligase (NEB). The ligation was transformed into DH5α cells via electroporation and the resulting colonies assessed for the presence of the *fts*Z insert via colony PCR using primers pBAD24_F and ECftsZ_R (Table 3), Taq polymerase and Thermopol buffer, under standard conditions (NEB).

**Table 3:** Primers used in this study.

Primer	Sequence
ECftsZ_F	5′-GCATGTCCCGGGATGTTTGAACCAATGGAACTT-3′
ECftsZ_R	5′-GCTATACTGCAGTTAATCAGCTTGCTTACGCA-3′
pBAD24_F	5′ - GCTAGCAGGAGGAATTCACC - 3′
pBAD24_R	5′ - GCCTGCAGGTCGACTCTAG - 3′

### Microscopic analysis of bacterial populations

Bacterial cells lengths were analysed using phase-contrast microscopy. Fixed cells were attached to poly-L-lysine coated slides and examined at 1000× magnification on a Zeiss Axioplan 2 microscope (Carl Zeiss, Jena, Germany). Cell length was manually measured for at least 100 cells per sample (except where indicated), using AxioVision 4.5 (Carl Zeiss), and the curve measuring tool.

### Induction of filamentation with cephalexin

Two flasks containing 200 mL each of LB media, with either 0 or 30 µg/ml cephalexin, were inoculated with an overnight culture of DH5α to give an initial OD_600_∼0.05. Cultures were incubated at 37°C with shaking at 150 rpm for 2 hours. Aliquots were removed and fixed in 4% formaldehyde at 1, 1.5 and 2 hours growth. Cell lengths were analysed via phase-contrast microscopy as described above. Live cell populations were generated using the same method, with the exception that cells were not fixed, but stored on ice after cephalexin exposure.

### Flow cytometry analysis and sorting

Flow cytometry analysis and sorting was carried out on the Aria II flow cytometer (BD Biosciences), at the Advanced Cytometry Facility at the Centenary Research Institute, Sydney, Australia. Cells were pelleted and resuspended in 1× PBS (filtered through a 0.2 µm filter) to an OD_600_ of 0.1–0.2 before analysis and sorting. In later experiments (sorting of the *E. coli* genomic library), cells were diluted directly into growth media (OD_600_ of 0.1–0.2) for analysis and sorting. Cultures were analysed at 15000–25000 events per second for initial sorts with the yield mask, and at 10–50 events per second for re-sorting with the purity mask. 1× PBS was used as sheath fluid, applied at a pressure of 70 psi, with a 70 µm nozzle for droplet formation and electrostatic charging for droplet sorting. Events were plotted on a log scale, with a window extension (WE) setting of 1, and thresholding on forward scatter (FSC) and side scatter (SSC) at 200. Signals for FSC and side scatter SSC, area (A), height (H) and width (W) were recorded. All sorts described below were from gates defined on a SSC-H (y axis) vs SSC-W (x axis) dot plot.

### Discrimination between short and filamentous cells

Populations with known cell-length distributions, (non-treated and cephalexin-treated, described above), were compared to determine which light scattering properties best corresponded to an increase in cell length. Populations of “short” or regular cell lengths (untreated DH5α cells), were compared to populations of increasing average cell length (exposed to cephalexin for 1, 1.5 and 2 hours) via flow cytometry.

### Isolation of filamentous cells from mixed populations using flow cytometry sorting

Mixed populations encompassing a range of cell lengths were generated by combining the non-cephalexin and cephalexin treated populations described above. Long or filamentous cells (>10 µm in length) were isolated from the mixed populations using flow cytometry sorting. Sorting gates were defined based on increasing SSC-W signal. Events in each gate were sorted using a yield mask (500 000 events collected per gate), and each sorted population was then re-sorted using a purity mask from the same gate (100 000 events collected per gate). Both sorted and re-sorted populations were concentrated using 0.2 µm spin filters (Millipore, Billerica, MA, USA), then attached to poly-L-lysine coated slides and analysed via phase-contrast microscopy as described above.

### Isolation of live, reproducibly filamentous clones from a mixed population using flow cytometry sorting

Filamentous EC766 (ftsZ*, P_BAD_) cells were sorted from a population of predominantly short EC764 (pBAD24) cells. EC766 (ftsZ*, P_BAD_), induced with 0.2% arabinose in M9 media, was used to spike a population of EC764 (pBAD24) at a ratio of 1∶100. Forty thousand events were sorted from the “filamentous” gate (yield mask), and 3000 events re-sorted from the “filamentous” gate (purity mask). The re-sorted population was plated onto LB agar with ampicillin at an expected density of 100 colony forming units (CFU) per plate, and incubated at 37°C overnight.

To determine whether the filamentous EC766 (ftsZ*, P_BAD_) cells had been recovered from the mixed population, 30 of the resulting colonies were randomly picked and subjected to colony PCR for the presence of the *fts*Z insert in the pBAD24 vector, using the primers pBAD24_F and ECftsZ_R as described above. Colonies that yielded a PCR product of the expected size (∼1 kb) were considered to contain the cloned *fts*Z gene insert, and were therefore considered to have been filamentous in the induced, mixed population. Cell viability of re-sorted filamentous cells was determined by comparing total number of colonies obtained to the total number of re-sorted events collected.

### Assessment of cell viability during flow cytometry sorting

To optimize cell viability during flow sorting, the handling of cells prior to, during and post sorting was altered. Cells were stored in M9 media prior to sorting, and sorted into M9 media. Additionally, cells were no longer stored on ice. The experiment described in the section above was repeated, with the addition of sorting and re-sorting events from the “short cell” gate. Both “short cell” and “long cell” re-sorted populations were plated onto LB with ampicillin, and incubated at 37°C overnight. Viability was determined as described above.

We also assessed viability with the Live/Dead *Bac*Light viability kit, as per the manufacturers instructions (Life Technologies, Carlsbad, CA, USA) to ascertain which stage of sorting process contributed to decreased cell viability. Cultures of short EC764 spiked with filamentous EC764 (as described in the previous section) were stained with 5 µM Syto9 (live stain) and 30 µM propidium iodide (dead stain). Live and dead control cells were prepared and stained according to the kit instructions, and run as single colour controls for colour compensation. Fluorescence was excited with a 488 nm blue laser, and detected with the 530 nm and 695 nm filters for Syto9 and propidium iodide respectively. Cultures were sorted as described above, and fresh Syto9 and propidium iodide was added to each sorted and resorted population to 5 µM and 30 µM, respectively. Populations were analysed to determine what proportion of cells stained as live or dead before and after each successive sort.

### Creation of a shotgun genomic expression library from *E. coli* DH5α

A shotgun genomic expression library was generated from *E. coli* DH5α genomic DNA cloned into the pBAD24 vector. Genomic DNA was extracted from an overnight culture using the Pure-Link Genomic DNA mini-kit (Life Technologies) according to the manufacturer's instructions. DNA was fragmented by partial restriction digestion with *Fat*I. DNA fragments between 1 and 5 kb were isolated with the QIAquick gel extraction kit (Qiagen,Hilden, Germany) according to the manufacturer's instructions. The vector pBAD24 was digested with *Nco*I and dephosphorylated with Antarctic phosphatase. Both vector and insert DNA were cleaned using a QIAquick PCR purification kit (Qiagen) as per the manufacturer's instructions. DNA concentration was determined on a Nanodrop (Thermofischer, Waltham, MA, USA), and confirmed by agarose gel electrophoresis. Cut and dephosphorylated pBAD24 was ligated to the prepared insert DNA T4 DNA ligase. Ligations were transformed into competent *E. coli* DH5α cells via electroporation, and clones selected on LB agar with ampicillin. The rate of insertion and insert sizes were estimated by colony PCR on 30 randomly selected colonies using the primers pBAD24_F and pBAD24_R with *Taq* polymerase and Thermopol buffer (NEB) under standard conditions. Only clones which gave a PCR product between 1 and 5 kb were considered to contain an insert. The formula P = 1−(1−f)^N^ [64], was used to calculate the probability that any given portion of the genome had been captured in the library, where f is the proportion of the genome contained within a clone, N is the number of clones, and P is the probability.

Colonies were pooled by re-suspension in LB media with ampicillin, and stored as frozen aliquots in 20% glycerol at −80°C.

### Screening of a shotgun expression genomic library for filamentous clones

Frozen aliquots of the *E. coli* DH5α genomic library, and the control EC764 (pBAD24) were used to inoculate overnight cultures in LB. Overnight cultures were used to inoculate M9 media and induced for over-expression as described above in growth conditions.

After induction, each culture was pelleted, washed in M9 media with ampicillin, and analysed on the flow cytometer. The induced control EC764 (pBAD24) population was compared with the induced library to determine whether an increase in events was observed in the “filamentous” gate. The induced library was screened for filamentous clones by sorting 20 000 events from the “filamentous” gate (yield mask), and re-sorting 3000 events (purity mask) from the same gate. Re-sorted cell populations were plated onto LB media with ampicillin at an expected density of 100 CFU per plate, and incubated at 37°C overnight.

### Confirmation of filamentous phenotype

Colonies isolated from the flow cytometry screen were picked into 96-well plates containing LB media with ampicillin, and incubated overnight at 37°C and 100 rpm. Overnight cultures were used to inoculate 96-well plates containing M9 media with ampicillin and 0.2% arabinose (w/v). Plates were incubated for 6 hours at 37°C and 100 rpm. Cells were fixed in 4% (v/v) formaldehyde, and stored at 4°C for microscopic analysis. Glycerol was added to overnight cultures to 20% (v/v), which were then stored at −80°C.

Fixed cells were examined under phase-contrast microscopy to determine whether filamentation had occurred. Clones that exhibited a filamentous phenotype (>10 µm cell length) were isolated from frozen stocks and plasmid DNA was extracted. Plasmids were transformed into a fresh host background, *E. coli* K12 MG1655, and assayed as described above to confirm a filamentous phenotype with over-expression of the cloned DNA. Plasmid DNA from clones with a reproducibly filamentous phenotype was sequenced from each end using the pBAD_F and pBAD_R primers (Table 3) to identify which genes or genomic regions were present in the cloned DNA.

### Assessment of false positive rate for induced libraries

EC764 (pBAD24) and DH5α pooled genomic library were grown under inducing conditions and screened as described above, with the exception that re-sorted filamentous cells were fixed immediately after re-sorting, concentrated and examined via phase contrast microscopy. Cell lengths were measured manually as described above, for as many cells as could be located on the microscope slide.

## Supporting Information

Figure S1 **The effect of cephalexin exposure on E. coli cell length.** (A) Average cell lengths of *E. coli* DH5α cells exposed to either none (0) or 30 µg/ml cephalexin for 1, 1.5 and 2 hours. Error bars show standard deviation. Cells were measured manually via phase contrast microscopy, and representative images of populations from each condition are shown in (B) no cephalexin, (C) 1 hour, (D) 1.5 hour and (E) 2 hours cephalexin exposure. Images were taken at 100× magnification, and scale bars = 10 µm.
(TIF)

Figure S2 **The effect of FtsZ over-expression on E. coli cell length.** (A) Cell length distributions of induced control EC764 (*E. coli* DH5α with pBAD24 vector) and EC766 (ftsZ*, P_BAD_) populations. Representative images of (B) induced control EC764 and (C) induced EC766. Cultures were induced in minimal media with 0.2% arabinose (w/v) for 3 hours. Images taken using phase contrast, scale bars = 10 µm
(TIF)
