# Supplementary figures and images for: Harnessing Single Cell Sorting to Identify Cell Division Genes and Regulators in Bacteria
Source: PLoS One. 2013 Apr 2;8(4):e60964. doi: 10.1371/journal.pone.0060964 (PMC3614548; doi:10.1371/journal.pone.0060964)

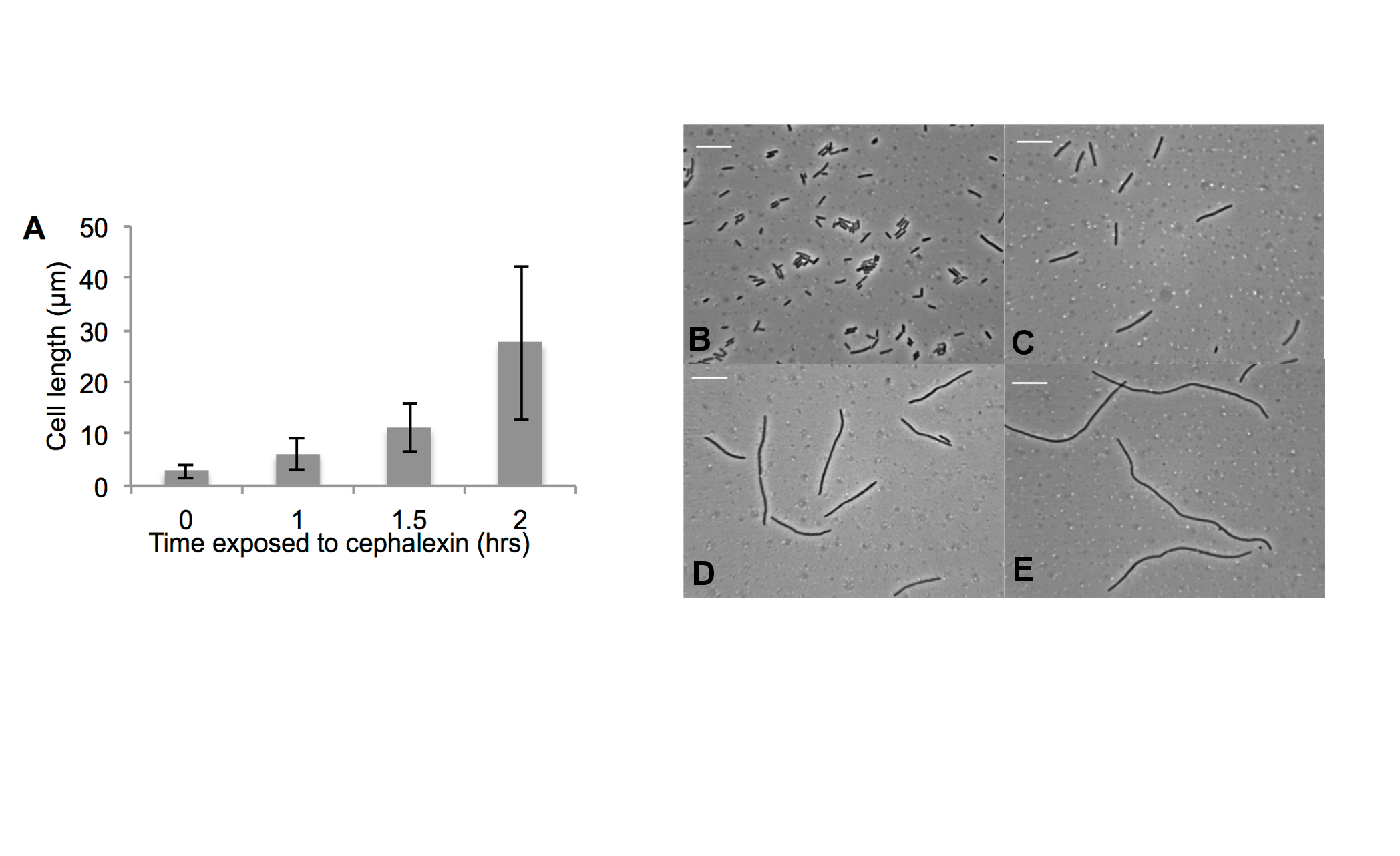

Supplement: Figure S1 — The effect of cephalexin exposure on E. coli cell length. (A) Average cell lengths of E. coli DH5α cells exposed to either none (0) or 30 µg/ml cephalexin for 1, 1.5 and 2 hours. Error bars show standard deviation. Cells were measured manually via phase contrast microscopy, and representative images of populations from each condition are shown in (B) no cephalexin, (C) 1 hour, (D) 1.5 hour and (E) 2 hours cephalexin exposure. Images were taken at 100× magnification, and scale bars = 10 µm. (TIF) [file pone.0060964.s001.tif]

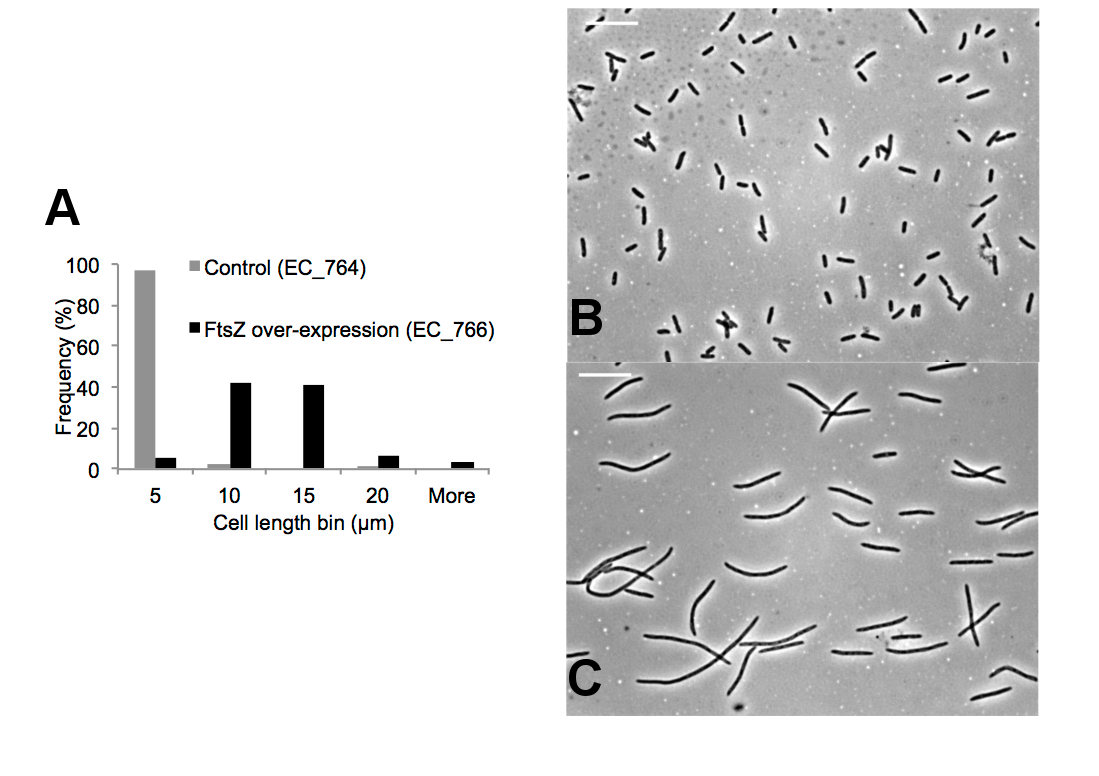

Supplement: Figure S2 — The effect of FtsZ over-expression on E. coli cell length. (A) Cell length distributions of induced control EC764 (E. coli DH5α with pBAD24 vector) and EC766 (ftsZ*, PBAD) populations. Representative images of (B) induced control EC764 and (C) induced EC766. Cultures were induced in minimal media with 0.2% arabinose (w/v) for 3 hours. Images taken using phase contrast, scale bars = 10 µm (TIF) [file pone.0060964.s002.tif]
